# Subacute combined degeneration of the spinal cord with cerebellar lesions: A case report

**DOI:** 10.1097/MD.0000000000037605

**Published:** 2024-05-24

**Authors:** Manmin Zhu, Changyin Yu, Zucai Xu, Haiqing Zhang, Hao Huang

**Affiliations:** a Department of Neurology, Affiliated Hospital of Zunyi Medical University, Zunyi, China.

**Keywords:** autoimmune gastritis, cerebellum, magnetic resonance imaging, subacute combined degeneration of the spinal cord, vitamin B12

## Abstract

**Rationale:**

Subacute combined degeneration of the spinal cord is a degenerative disease of the central and peripheral nervous systems caused by vitamin B12 deficiency, mainly involving the spinal cord posterior, lateral, and peripheral nerves, but rarely involving the cerebellum.

**Patient concerns:**

A 41-year-old woman presented with a 2-year history of walking unsteadily. Her hematologic examination revealed megaloblastic anemia and vitamin B12 deficiency. Electromyography showed multiple peripheral nerve damage (sensory fibers and motor fibers were involved). Imaging examination showed long T2 signal in the cervical, thoracic and lumbar spinal cord and cerebellum. Gastroscopy revealed autoimmune gastritis.

**Diagnoses:**

Subacute combined degeneration of the spinal cord.

**Interventions:**

By supplementing with vitamin B12.

**Outcomes:**

The patient’s symptoms of limb weakness, diet, and consciousness were improved, and the muscle strength of both lower limbs recovered to grade IV.

**Lessons:**

The symptomatic people should seek medical treatment in time to avoid further deterioration of the disease. When esophagogastroduodenoscopy is performed as part of routine physical examination in asymptomatic people, it should be checked for the presence of autoimmune gastritis. Early diagnosis can prevent irreversible neuropathy.

## 1. Introduction

Subacute combined degeneration of the spinal cord is a common neurological disease associated with vitamin B12 deficiency, with clinical manifestations of gastrointestinal, blood, nervous and psychiatric system abnormalities, mainly involving the spinal cord posterior, lateral and peripheral nerves, but rarely involving the cerebellum. This study reports the diagnosis and treatment of a patient with subacute combined degeneration of the spinal cord and cerebellar lesions admitted to the Department of Neurology, the Affiliated Hospital of Zunyi Medical University, aiming to enhance the clinical cognition of neurologists of the disease, so as to prevent the disease progression and adverse clinical outcomes.

## 2. Case report

A 41-year-old woman presented with progressive weakness of her lower limbs and even difficulty in walking due to poor diet 2 years ago. Gastroscopy showed “autoimmune gastritis, multiple gastric polyps (Yamada type I),” and “gastric polypectomy” was performed. Six months ago, the patient’s diet became worse again after she was infected with the novel coronavirus, and the weakness of both lower limbs aggravated to the point that she could not stand and walk, accompanied by slow reaction, vague speech, involuntary shaking of his hands, and difficulty in holding objects. After admission on March 3, 2023, physical examination showed that the temperature was 36.5°C, the pulse was 82 beats/min, the respiration was 20 breaths/min, and the blood pressure was 106/62 mm Hg (1 mm Hg = 0.133 kPa). She had normal development, anemic appearance, poor skin elasticity, slightly yellowish sclera, and pale lips. Nervous system examination showed tongue extension slightly deviation to the left, no atrophy of limb muscles, grade IV muscle strength of both upper limbs, grade III muscle strength of lower limbs, physiological reflex of limbs was not evoked, deep sensory disturbance of lower limbs, finger-nose test was not correct, bilateral Hoffmannn sign (-), Chaddock sign (-), Babinski sign (-).

Laboratory tests showed hemoglobin 70.0 g/L, hematocrit 0.19 L/L, mean corpuscular volume 124.0 fL, and mean corpuscular hemoglobin 45.5 pg. The reticulocyte absolute value was 186.8 × 10^9^/L, the reticulocyte percentage was 9.7%, the reticulocyte percentage was 9.0%, the reticulocyte percentage was 20.9%, and the reticulocyte percentage was 70.1%. Vitamin B12: <50 pg/mL (reference range: 180–914 pg/mL); two kinds of atrophic gastritis antibodies: anti-intrinsic factor antibody IgG was negative, anti-parietal cell antibody IgG was positive; Gastrin level >1000.00 pg/mL (reference range: 13.00–115.00 pg/mL). Examination of the cerebrospinal fluid was normal.

**Figure 1.**
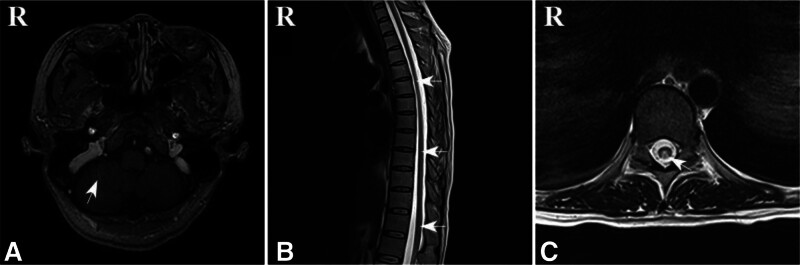
(A) Slightly hyperintense T2 signal was seen in the cerebellar hemisphere, and no abnormal signal areas were seen in the rest of the brain parenchyma. (B) The contours of the cervical and thoracic spinal cord were blurred, and the signal intensity of the dorsal spinal cord was uneven, like a slightly long T2 signal band. Long T2 signal in the posterior cord of the thoracic and lumbar spinal cord. (C) An inverted “V” sign was seen in the posterior cord of the thoracolumbar spinal cord.

Brain magnetic resonance imaging (MRI) plain scan and enhancement: long T2 signal in the cerebellar hemisphere, linear enhancement shadow in the cerebellar hemisphere (Fig. 1A). MRI scan of the cervical spine: the outline of the cervical and thoracic spinal cord was blurred, the signal intensity of the dorsal spinal cord was uneven, and the signal intensity was slightly longer in a band-like manner (Fig. 1B). MRI of the thoracic spine showed long T2 signal in the posterior cord of the thoracolumbar spinal cord (Fig. 1B), showing an inverted “V” sign (Fig. 1C). Electromyography showed multiple peripheral nerve damage (sensory fibers and motor fibers were involved).

Combined with the patient’s symptoms, signs, and related examination results, the clinical symptoms were significantly improved after vitamin B12 supplementation, so the diagnosis of subacute combined degeneration of the spinal cord caused by vitamin B12 deficiency was confirmed. After 2 weeks of vitamin B12 supplementation, the patient’s symptoms of limb weakness, diet, and consciousness were improved, and the muscle strength of both lower limbs recovered to grade IV, and she was discharged from the hospital on March 17, 2023. After discharge, the patient was advised to continue vitamin supplementation (vitamin B12 injection 1 mg/time, once a day intramuscular injection, adjusted to 2–3 times a week after 3 months; vitamin B12 tablets 1000–1500 µg/time, oral once a day; mecobalamin tablets 0.5 mg/time, oral 3 times a day to promote the absorption of B vitamins in the body; and increase the intake of lean meat and protein foods).

## 3. Conclusions

As a coenzyme in the process of nucleoprotein synthesis and myelination, vitamin B12 plays an important role in myelin synthesis in the central nervous system.^[1]^ Any abnormality in the uptake, absorption, combination, and utilization of vitamin B12 can lead to vitamin B12 deficiency. At present, the main causes of vitamin B12 deficiency are the following^[2,3]^: insufficient intake, such as strict vegetarians; malabsorption, in patients with autoimmune gastritis (pernicious anemia), celiac disease, inflammatory bowel disease, surgical gastrectomy, gastric bypass, and ileal resection; impaired binding of vitamin B12 to intrinsic factors (such as anti-intrinsic factor antibody and anti-parietal cell antibody); deficiency of vitamin B12 utilization and congenital deficiency of vitamin B12 metabolism; drug interference, such as metformin and proton pump inhibitors.

Subacute combined degeneration of the spinal cord is a degenerative disease of the central and peripheral nervous systems caused by vitamin B12 deficiency, involving the posterior and lateral funiculus of the spinal cord and peripheral nerves. The main clinical symptoms were walking instability, difficulty standing with eyes closed, numbness of both lower limbs or limbs, feeling of stepping on cotton, and limb tingling, which were often the early manifestations of subacute combined degeneration of the spinal cord. Studies of the cerebrospinal fluid are usually normal. MRI of the lower cervical and upper thoracic spinal cord usually shows strip and speckled lesions with low T1 signal and high T2 signal. In electromyography, both sensory and motor nerves were damaged, mainly accompanied by demyelinating lesions and partial axonal damage.^[4]^

In this case, in addition to the common neurologic manifestations mentioned above, such as deep sensory impairment, peripheral nerve dysfunction, and cognitive dysfunction, the patient also had significant symptoms and signs of cerebellar dysfunction. Previous studies have found that nervous system lesions caused by vitamin B12 deficiency mostly involve the posterior funiculus of the cervicothoracic spinal cord,^[5]^ and rarely involve the cerebellum. Because the cerebellum has less requirement for vitamin B12 and less storage, it is more likely to have cerebellar damage once vitamin B12 is deficient for a long time.^[6,7]^ Studies have confirmed that long-term administration of mecobalamin (a vitamin B12 analogue) can protect rat cortical neurons from N-methyl-D-aspartate receptor-mediated neurotoxicity,^[8]^ and can reduce the damage of neurotoxicity to mouse cerebellar granule cells.^[9]^ In addition, vitamin B12 can also promote the growth of the neurite of mammalian cerebellar granule neurons.^[10]^ This suggests that vitamin B12 not only protects against cerebellar neurotoxicity, but also is required for cerebellar nerve development. The clinical symptoms caused by cerebellar lesions were significantly relieved, suggesting that cerebellar lesions are similar to other nervous system lesions. Early treatment after onset can reverse the severity of the disease to a certain extent, so timely diagnosis is important. Unfortunately, we did not repeat MRI to assess the changes in brain and spinal cord lesions after treatment.

In short, in addition to the common neurological symptoms, subacute combined degeneration of the spinal cord may also present with rare symptoms such as cerebellar dysfunction. At the same time, clinicians should be alert to the possibility of vitamin B12 deficiency in patients with cerebellar ataxia, so that timely diagnosis, standardized treatment and better prognosis can be achieved.

## Author contributions

**Writing – original draft, writing – review & editing:** Manmin Zhu.

**Funding acquisition:** Changyin Yu, Zucai Xu, Haiqing Zhang, Hao Huang.

**Supervision:** Changyin Yu, Zucai Xu.

**Conceptualization:** Haiqing Zhang, Hao Huang.

**Data curation:** Haiqing Zhang.

**Formal analysis:** Hao Huang.
